# Supporting social-emotional and behavioral skills in preschool children through gymnastics

**DOI:** 10.1186/s12887-026-07204-8

**Published:** 2026-06-25

**Authors:** Emre Yamaner, Özlem Bal, Merve Girgin, Kerim Aktaş, Nisa Gökden Kaya, Fatime Sabahat Işıktekiner

**Affiliations:** 1 Child and Youth Services Department, Hitit University, Sungurlu Vocational College, Corum, Turkey; 2 Faculty of Health Sciences, Child Development Department, Hitit University, Corum, Turkey; 3 Faculty of Health Sciences/College, Child Development Department, Istanbul Medipol University, Istanbul, Turkey

**Keywords:** Preschool children, Gymnastics, Social-emotional development, Self-regulation, Mixed-method design

## Abstract

**Background:**

This study aimed to evaluate the effects of a structured basic gymnastics education program on the social-emotional and behavioral skills of preschool children. The study sample consisted of 40 typically developing children aged 48–69 months who were enrolled in private early childhood education institutions in Çorum province, Türkiye.

**Methods:**

A mixed-method research design was employed, integrating quantitative and qualitative approaches. The quantitative component adopted a pretest–posttest control group quasi-experimental design, whereas the qualitative component was conducted using a case study approach. The experimental group (*n* = 20) participated in an eight-week structured basic gymnastics education program, whereas the control group (*n* = 20) continued with the regular preschool curriculum without additional intervention. Group assignment was based on existing classroom structure. Quantitative data were collected through teacher-reported measures, including the Self-Regulation Skills Scale, the Emotion Regulation Scale, and the Social Competence and Behavior Assessment Scale. Qualitative data were obtained via semi-structured interviews with parents. Quantitative analyses were performed using SPSS software. Data normality was examined using the Shapiro–Wilk test, and depending on distributional assumptions, independent samples t-tests, Mann–Whitney U tests, and Wilcoxon signed-rank tests were applied. Effect sizes were calculated using Cohen’s d for parametric analyses and r coefficients for nonparametric analyses.

**Results:**

Quantitative findings indicated no statistically significant differences between the experimental and control groups at baseline across all outcome variables (all *p* > .05; d < 0.20), confirming baseline comparability. Posttest comparisons revealed that children in the experimental group achieved significantly higher scores in self-regulation, emotion regulation, and social competence compared to those in the control group (all *p* < .05). The associated effect sizes ranged from moderate to large (*r* = .46–0.82), indicating meaningful intervention effects. Within-group analyses further demonstrated significant and substantial improvements across all measured variables in the experimental group from pretest to posttest (all *p* < .05; *r* > .80). Consistent with these quantitative findings, qualitative analyses identified themes reflecting notable improvements in children’s emotional awareness, self-control, social interaction, and behavioral regulation skills.

**Conclusions:**

The findings suggest that basic gymnastics education represents an effective intervention for supporting the social-emotional and behavioral development of preschool children.

**Graphical Abstract:**

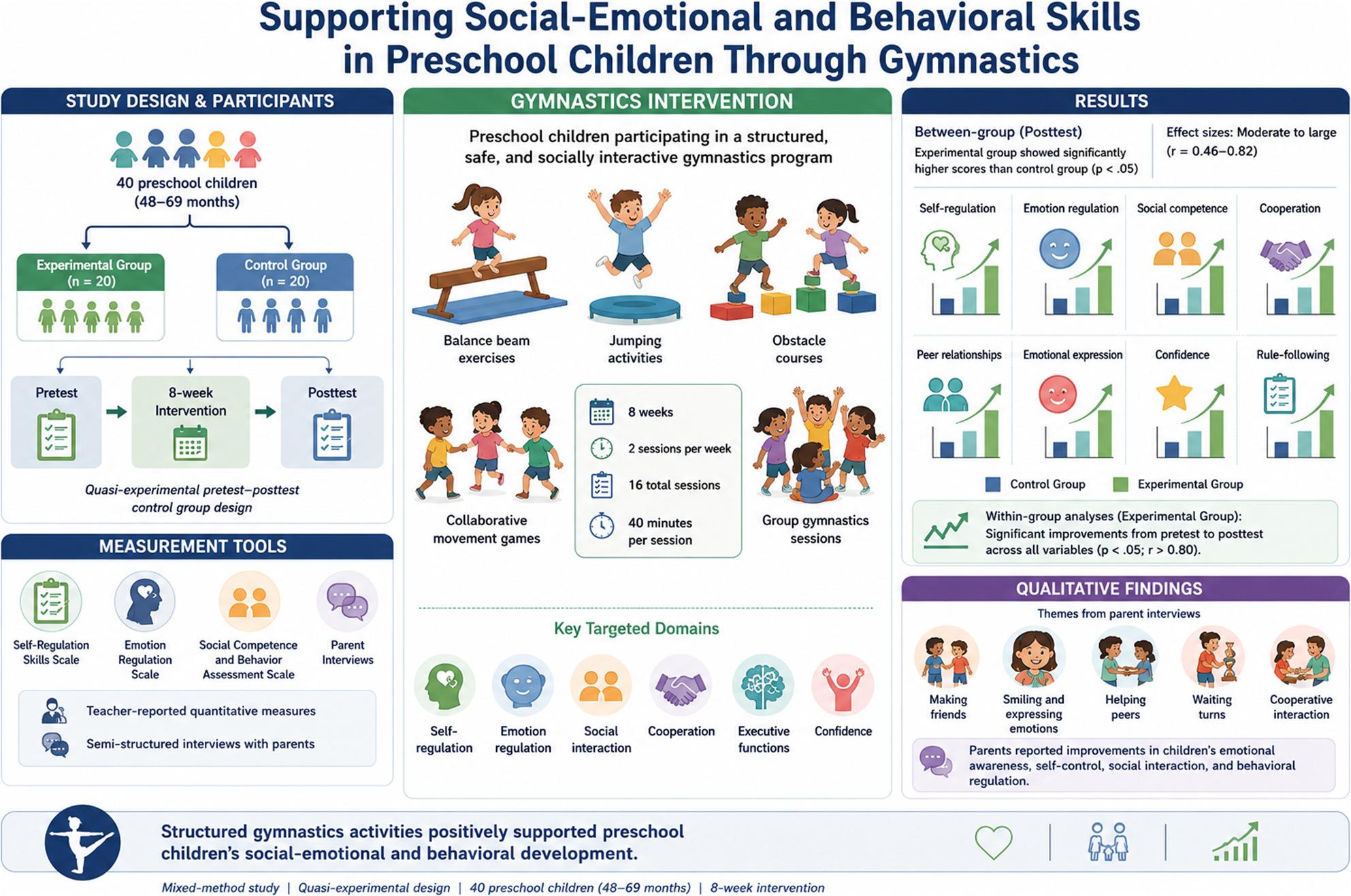

**Supplementary Information:**

The online version contains supplementary material available at 10.1186/s12887-026-07204-8.

## Introduction

 Early childhood is a critical period in terms of social-emotional development [1]. Foundational theories of child development, particularly those proposed by Piaget and Vygotsky, emphasize the critical role of early childhood in shaping developmental trajectories. Piaget conceptualized development as a dynamic process arising from both biological maturation and active interaction with the environment. Although his theoretical framework primarily addressed cognitive development, Piaget also highlighted the role of play and collaborative activities in fostering social development. In parallel, Vygotsky’s sociocultural theory underscores social interaction as a central mechanism of learning and development, particularly through the zone of proximal development, which reflects the importance of guided participation and shared activities. Complementing these theoretical perspectives, the Collaborative for Academic, Social, and Emotional Learning (CASEL), founded in 1994, conceptualizes Social–Emotional Learning (SEL) as an integrative framework encompassing cognitive, emotional, and social domains, emphasizing that academic achievement is closely linked to students’ emotional well-being and interpersonal skills [2].

Supporting children’s social and emotional development during early childhood plays a critical role in the acquisition of foundational social skills [2]. These skills encompass abilities such as active listening, empathy, appropriate self-expression, problem solving, and effective participation in group activities [3]. Psychosocial development is influenced by a wide range of factors [4, 5], among which participation in sports and physical activity has been identified as a particularly salient contributor [6–8]. Engagement in sports activities has been shown to foster essential interpersonal and intrapersonal competencies, including cooperation, teamwork, responsibility, leadership, emotional regulation, respect for others, and social acceptance, while also enhancing children’s capacity to observe and adapt to social contexts [9, 10].

Among the physical activities that support child development, gymnastics has been identified as a particularly influential discipline [11, 12]. Beyond its well-established contributions to motor development [13], gymnastics also plays a significant role in social-emotional development by promoting communication during group activities, fostering appropriate responses to success and failure, reinforcing perseverance, and encouraging rule adherence and respect for others [11]. Emotion regulation, defined as the ability to shape, evaluate, and monitor one’s emotional experiences, constitutes a core component of social-emotional development [14]. Accumulating evidence indicates that participation in physical activity is positively associated with emotion regulation capacities in children [15, 16]. These skills are essential for managing interpersonal conflicts and adapting to the social and environmental demands encountered during early childhood [17–19].

Self-regulation refers to the capacity to intentionally control and modulate one’s thoughts, emotions, behaviors, impulses, and attentional processes through cognitive strategies [20, 21]. Empirical evidence indicates that self-regulation is shaped by multiple contextual and individual factors, including engagement in physical activity [22, 23], parental education level [24], role modeling [25], parental attitudes [26], peers’ self-regulatory behaviors [27], and broader cultural influences [28].

Social competence represents another key domain that predicts self-regulation skills [29], and evidence suggests that social competence and emotion regulation are closely interconnected developmental constructs [30, 31]. Social competence enables children to participate effectively in social contexts by encompassing abilities such as emotional control, prosocial behavior, and adaptive interaction, all of which are critical for successful social adjustment [32]. Within this framework, gymnastics has been identified as a physical activity that facilitates the development of skills relevant to social competence [12]. Empirical findings further indicate that participation in gymnastics and sports activities during early childhood contributes positively to a wide range of motor outcomes, including jumping ability [33], agility, flexibility, vertical and standing long jumps [34], running, throwing, forward bending, balance [35, 36], speed, grip strength [37], overall physical fitness [38], locomotor skills, and object control [39, 40]. Beyond motor development, engagement in sports activities has also been associated with improvements in peer relationships, social acceptance, sense of belonging, adaptive behaviors, rule-following, and school satisfaction [41, 42].

Despite the growing body of literature demonstrating the benefits of physical activity and gymnastics for motor and selected psychosocial outcomes in early childhood, existing studies have largely examined these effects in isolation or through single-domain approaches. In particular, there is a lack of experimentally controlled, multidimensional intervention studies that simultaneously investigate self-regulation, emotion regulation, and social competence within a unified gymnastics-based framework. Moreover, much of the previous research has relied on descriptive or correlational designs, which limits causal interpretations regarding developmental change over time. Consequently, it remains unclear whether structured basic gymnastics education leads to differential improvements in social-emotional and behavioral outcomes beyond those associated with typical preschool education. Addressing this gap, the present mixed-method study employs a pretest–posttest control group design to examine the effects of an eight-week basic gymnastics education program on preschool children’s self-regulation, emotion regulation, and social competence. Research Questions and Hypotheses.

The present study aimed to examine the effects of an eight-week basic gymnastics education program on preschool children’s social-emotional and behavioral development using a mixed-method approach.

For the quantitative strand, the following hypotheses were tested:

- H1: Children in the experimental group who participated in the basic gymnastics education program will demonstrate significantly greater improvements in self-regulation skills from pretest to posttest compared to children in the control group (group × time effect).
- H2: Children in the experimental group will show significantly greater improvements in emotion regulation skills over time compared to children in the control group.
- H3: Children in the experimental group will exhibit significantly greater increases in social competence and significantly greater reductions in maladaptive behavioral indicators (anger–aggression and anxiety–introversion) compared to the control group from pretest to posttest.

For the qualitative strand, the study explored parents’ interpretations of the effects of the basic gymnastics’ education program on their children’s social-emotional and behavioral development.

## Method

The study was conducted using a mixed-method design integrating quantitative and qualitative approaches, enabling a more comprehensive analysis and a deeper understanding of the research problem [43]. Specifically, a sequential explanatory mixed-method design (QUAN qual) was employed. In this design, quantitative data were collected and analyzed first, followed by qualitative data collection to further explain and elaborate the quantitative findings.

The quantitative component followed a pretest–posttest control group quasi-experimental design. Prior to the intervention, all children in both the experimental (*n* = 20) and control (*n* = 20) groups were assessed using standardized teacher-report scales measuring self-regulation, emotion regulation, and social competence. Following the pretest assessments, the experimental group participated in an eight-week Basic Gymnastics Education Program implemented by a sports scientist who was also the primary researcher (see Appendix, Table 5 and Table 6). To ensure the fidelity and internal validity of the intervention, the process was conducted in accordance with a pre-structured and standardized training protocol. This protocol included detailed guidelines regarding exercise selection, duration, intensity, rest intervals, and progression principles. Prior to the intervention, participants were introduced to the exercises and taught proper movement patterns appropriate for their age. During the intervention, adherence to the protocol was continuously monitored, and training logs were systematically maintained to ensure consistent participation. Additionally, all sessions were conducted at similar times of day to minimize the potential effects of circadian variations. While the control group continued with their regular preschool curriculum, no additional intervention was implemented. This study employed a quasi-experimental design. The research was conducted in a preschool setting deemed appropriate for the study objectives in terms of accessibility and implementation feasibility. Due to practical considerations, including ensuring full participation and accessibility of children, one intact classroom attending in the morning session was assigned as the experimental group, while the control group consisted of children attending in the afternoon session. This temporal separation was also intended to reduce potential interaction and contamination between groups. Therefore, no randomisation procedure was applied, and group assignment was based on existing classroom structure. After the intervention period, posttest assessments were administered to both groups using the same instruments. The outcome measures were based on teacher-report scales that require evaluation of children’s behaviors through natural classroom observations. Therefore, each classroom teacher assessed the children in their own class (experimental or control group). Given the study design, teachers were aware of group allocation, and thus complete blinding was not feasible. Nevertheless, to reduce potential evaluation bias, teachers were not informed about the specific hypotheses or expected direction of the results. The qualitative component was designed as a case study. After the completion of the posttest assessments, qualitative data were collected through semi-structured interviews with parents of children in the experimental group in order to evaluate the perceived effectiveness of the gymnastics program. The interviews were conducted by two trained undergraduate interns who were not involved in the quantitative assessment process. The use of open-ended questions allowed flexibility in the interview process and enabled participants to express their perspectives and experiences in depth [44].

Integration of quantitative and qualitative data occurred at the interpretation stage. A connecting strategy was used, whereby the quantitative results informed the focus of the qualitative inquiry. Qualitative themes were systematically compared with quantitative outcomes to examine convergence, complementarity, or divergence. This integration enabled a comprehensive understanding of the effectiveness of the Basic Gymnastics Education Program.

### Study group

The study sample was determined using purposive sampling and consisted of 40 typically developing children aged 48–69 months who were enrolled in a private kindergarten in Çorum province. The experimental group included 20 children (11 girls, 9 boys), and the control group included 20 children (12 girls, 8 boys). The children came from families with middle socioeconomic status. To determine the appropriate sample size, an a priori power analysis was conducted using G*Power version 3.1, based on a one-tailed t-test for two independent groups. Based on previous literature [12], the expected effect size was set at d = 0.74. The analysis was performed with a significance level of α = 0.05, statistical power of 1 − β = 0.72, and an allocation ratio of 1:1 between groups. The results indicated that a minimum of 19 participants per group (total *n* = 38) was required. To account for potential data loss and attrition, 20 participants were included in each group, resulting in a total sample size of 40 children.

### Data collection tools

#### Self-regulation skills scale

The Self-Regulation Skills Scale, developed by Bayındır and Ural [45], consists of 33 items and comprises two subdimensions: regulation skills and control skills. The regulation skills subscale includes 21 items assessing planning, process monitoring, action control, evaluation, emotional regulation, and motivational regulation, whereas the control skills subscale consists of 12 items measuring self-control and attentional control. The scale is a 5-point Likert-type instrument, ranging from 1 (definitely not true) to 5 (definitely true). Scale scores are calculated by dividing the total score by the number of items. The content validity index for the overall scale was reported as 0.78, and the overall reliability coefficient was 0.96. Internal consistency coefficients were 0.96 for the regulation skills subscale and 0.91 for the control skills subscale, indicating that the scale demonstrates strong validity and reliability [45].

#### Emotion regulation scale

The Emotion Regulation Scale was developed by Shields and Cicchetti [46] and adapted into Turkish by Batum and Yağmurlu [47]. The scale is designed to assess preschool children’s emotional reactivity and their capacity to modulate and express emotions across different contexts. It consists of 24 items organized into two subscales: Emotion Regulation and Variability–Negativity. The instrument is a 4-point Likert-type scale completed by teachers or parents, with response options ranging from 1 to 4. The internal consistency coefficient for the teacher-reported form was reported as 0.75, indicating acceptable reliability [47].

#### Social competence and behavior assessment scale-30

The Social Competence and Behavior Assessment Scale (SCBE-30) was developed by LaFreniere and Dumas [48], and its Turkish adaptation and standardization were conducted by Çorapçı et al. [49]. The scale consists of 30 items designed to assess children’s social competence and behavioral adjustment across three subscales: Social Competence (10 items), Anger–Aggression (10 items), and Anxiety–Introversion (10 items). The Social Competence subscale evaluates cooperative behaviors and problem-solving skills; the Anger–Aggression subscale assesses externalizing behaviors, including defiance toward adults and aggressive interactions with peers; and the Anxiety–Introversion subscale measures internalizing behaviors such as negative emotionality and lack of initiative. The instrument is rated on a 6-point Likert scale, with response options ranging from 1 (never) to 6 (always), reflecting the frequency of the observed behaviors [49].

#### Parent interview form

The interview form was developed by the researchers based on expert consultation with five specialists, including one expert in sports science, three experts in preschool education, and one preschool teacher. The form consisted of six open-ended questions designed to elicit parents’ perspectives on the effects of gymnastics activities on children’s emotional and behavioral regulation skills, as well as their social skills.

### Data analysis

The study employed a mixed-method design integrating quantitative and qualitative approaches, allowing for a more comprehensive analysis and a more accurate understanding of the research problem [43]. The qualitative component was designed as a case study, whereas the quantitative component followed a pretest–posttest control group quasi-experimental design. Children were assigned to the experimental and control groups based on existing classroom structure. The dependent variables were children’s levels of self-regulation, emotion regulation, and social competence, while the independent variable was the Basic Gymnastics Education Program implemented with the experimental group for a period of eight weeks.

Qualitative data were collected through semi-structured interviews with parents to evaluate the perceived effectiveness of the gymnastics program. The use of open-ended questions provided flexibility during the interview process and enabled participants to articulate their perspectives and experiences in depth [44].

Prior to quantitative analyses, data normality was assessed using the Shapiro–Wilk test. As most variables did not meet the normality assumption (*p* < .05), nonparametric statistical tests were employed for the primary analyses. Mann–Whitney U tests were used for between-group comparisons of posttest scores, while Wilcoxon signed-rank tests were conducted to examine within-group pretest–posttest differences. For variables that met the normality assumption in baseline comparisons between the experimental and control groups, independent samples t-tests were applied. Effect sizes were calculated to estimate the magnitude of observed differences, using Cohen’s d for parametric analyses and r coefficients for nonparametric analyses [50]. Effect sizes were interpreted as small (d = 0.20; *r* = .10), medium (d = 0.50; *r* = .30), and large (d = 0.80; *r* = .50) [50, 51]. Statistical significance was set at *p* < .05.

Qualitative data were analyzed using content analysis. Parents’ responses were grouped based on conceptual similarity and systematically classified into themes and subthemes [52]. To ensure confidentiality, direct quotations were anonymized and participants were coded as E1 through E20. To enhance the reliability of the qualitative analysis, the data were independently coded by a second researcher. All interview participants were mothers, as fathers did not volunteer to participate in the interview process.

During the coding process, one coder initially identified 22 items, while the second coder identified 23 items. Following a systematic comparison and consensus-building procedure, the number of items was refined to 20. Agreement was reached on 17 of these items, yielding an inter-coder agreement rate of 85%, which is considered acceptable and sufficient for qualitative research. Inter-coder reliability was calculated using the formula: Reliability = Agreement / (Agreement + Disagreement) × 100 [53].

### Findings

The distributional properties of pretest and posttest scores for both the experimental and control groups were examined using the Shapiro–Wilk test. The results indicated that several subdimensions did not meet the assumption of normality (*p* < .05); therefore, parametric or nonparametric statistical tests were applied as appropriate. The study findings are presented under two main sections: quantitative findings and qualitative findings.

#### Quantitative findings

Comparisons of pretest scores across all outcome variables revealed no statistically significant differences between the experimental and control groups (all *p* > .05). Consistently small Cohen’s d effect sizes (d < 0.20) further confirmed that the groups were comparable at baseline, indicating similar initial characteristics at the outset of the study (Table 1).

**Table 1:** Between-group comparison results of pre-test and post-test scores of the experimental and control groups

			Pre-test				Post-test			
Scale	Group	*N*	x̅±Sd	t	*p*	Cohen’s d	Median(IQR)	U	*p*	r
RS	Intervention	20	56.50±9.22	-0.51	0.611	0.16	90 (3)	31.5	<.001*	0.78
	Control	20	57.85±7.32				57.5 (12.25)			
CS	Intervention	20	33.50±4.92	-0.61	0.547	0.19	55 (2.75)	22.5	<.001*	0.821
	Control	20	34.35±3.86				35 (6.75)			
VN	Intervention	20	43.45±5.61	0.47	0.642	0.15	22 (8.75)	94	.003*	0.459
	Control	20	42.65±5.18				43 (8.75)			
ER	Intervention	20	20.45±3.12	0.11	0.91	0.04	31 (2.75)	88.5	.002*	0.487
	Control	20	20.35±2.37				20 (3)			
SC	Intervention	20	31.00±7.15	-0.08	0.94	0.02	50 (9)	40	<.001*	0.731
	Control	20	31.15±5.12				40 (0)			
A-A	Intervention	20	29.50±5.57	0.13	0.898	0.04	10 (4)	61	<.001*	0.659
	Control	20	29.30±4.17				17 (7)			
A-I	Intervention	20	36.00±4.93	0.76	0.451	0.24	10 (7)	35	<.001*	0.748
	Control	20	34.95±3.69				36 (7.75)			

*RS* Regulation Skills. *CS* Control Skills, *VN* Variability–Negativity, *ER * Emotion Regulation, *SC* Social Competence, *A-A* Anger–Aggression, *A-I* Anxiety–Introversion. Independent samples t-tests and Cohen’s d effect sizes were used for pretest comparisons. As the normality assumption was not met for posttest comparisons, Mann–Whitney U tests were applied and effect sizes were reported using the r coefficient. Statistical significance was set at *p* < .05

Posttest comparisons demonstrated that the experimental group exhibited higher median scores across all variables compared to the control group, with these differences reaching statistical significance (*p* < .05). Effect sizes, calculated using the r coefficient, ranged from medium to predominantly large (*r* = .46–0.82), suggesting that the observed group differences were not only statistically significant but also of practical relevance (Table 1).

As presented in Table 1, no statistically significant differences were observed between the experimental and control groups at pretest across all variables (all *p* > .05), and the small effect sizes indicate that the groups were comparable at baseline. In contrast, posttest comparisons revealed statistically significant differences between the groups in favor of the experimental group (all *p* < .05). Children who participated in the intervention demonstrated superior social-emotional and behavioral outcomes compared to those in the control group. The effect sizes ranged from moderate to large, suggesting that the intervention exerted a meaningful and practically significant impact on children’s development.

Within-group pretest–posttest comparisons revealed statistically significant changes across all variables in both the experimental and control groups (all *p* < .05). The effect sizes calculated for within-group analyses were consistently large for all outcome measures (*r* > .80) (Table 2). The similarity observed in effect size values may be attributed to the constant sample size across groups and the comparable magnitude of the corresponding Z statistics (Table 2).

**Table 2:** Within-group comparisons of pretest and posttest scores in the experimental and control groups

			Pre-test	Post-test			
Scale	Group	*N*	Median (IQR)	Median (IQR)	Z	*p*	r
RS	Intervention	20	81 (10.75)	90 (3)	3.922	< 0.001*	0.877
	Control	20	58.4 (9.5)	57.5 (12.25)	3.921	< 0.001*	0.877
CS	Intervention	20	46 (6.25)	55 (2.75)	3.925	< 0.001*	0.878
	Control	20	35 (3.75)	35 (6.75)	3.932	< 0.001*	0.879
V/N	Intervention	20	30 (1)	22 (8.75)	-3.928	< 0.001*	0.878
	Control	20	41 (5)	43 (8.75)	-4.051	< 0.001*	0.906
ER	Intervention	20	29 (2)	31 (2.75)	3.930	< 0.001*	0.879
	Control	20	21 (2.75)	20 (3)	3.953	< 0.001*	0.884
SC	Intervention	20	31 (3.5)	50 (9)	4.028	< 0.001*	0.901
	Control	20	30 (3.5)	40 (0)	3.946	< 0.001*	0.882
A-A	Intervention	20	28 (6)	10 (4)	-3.937	< 0.001*	0.880
	Control	20	28 (5.5)	17 (7)	-3.947	< 0.001	0.882
A-I	Intervention	20	21.5 (7.75)	10 (7)	-3.924	< 0.001*	0.878
	Control	20	35 (6.5)	36 (7.75)	-3.929	< 0.001*	0.879

#### Qualitative findings

Parents of children in the experimental group were asked to report their perceptions of the effects of participation in gymnastics activities on their children’s social, emotional, and behavioral skills. Analysis of parental responses yielded two overarching themes: emotion and behavior. The emotion theme comprised the subthemes of anger management, coping with disappointment, self-confidence, and emotional expression. The behavior theme included the subthemes of sharing, helping, cooperation, waiting in line, patience, making friends, and rule-following. The qualitative findings corresponding to these themes are presented in Table 3.

**Table 3:** The effect of gymnastics training on children’s social-emotional skills

Theme	Sub-Theme	Number of Children
Emotion	Anger Management	4
	Coping with Disappointment	2
	Self-confidence	3
	Expressing Emotions	10
Behavior	Sharing	5
	Helping	4
	Cooperation	3
	Waiting in Line	4
	Patience	4
	Making Friends	11
	Following Rules	7
No change	I did not observe any change	2

As presented in Table 3, parents reported that participation in gymnastics activities contributed notably to children’s social-emotional development, particularly in relation to making friends and expressing emotions. Several parents highlighted observable improvements in peer interactions and emotional regulation. For instance, one parent E-5 noted that the child learned to share with peers and that previously observed aggressive behaviors had disappeared. Similarly, another parent E-11 reported improvements in social skills, emphasizing a transition from introverted and shy behavior toward greater ease in making friends. Additional accounts indicated enhanced coping with disappointment E-14, increased perseverance and self-efficacy when facing challenges E-18, and improved anger control following participation in gymnastics activities E-20.

Parents were subsequently asked to describe their children’s emotional and behavioral responses to gymnastics training. Analysis of the responses yielded two main themes: emotion and behavior. The emotion theme comprised the subthemes of excitement and cheerfulness, whereas the behavior theme included the subthemes of willingness and being energetic. The qualitative findings related to these themes are presented in Table 4.

**Table 4:** The children’s emotions and behaviors towards gymnastics training

Theme	Sub-Theme	Number of Children
Emotion	Excitement	8
	Cheerfulness	22
Behavior	Willingness	8
	Being Energetic	5

As shown in Table 4, parents reported that most children displayed positive emotional responses toward gymnastics training, with cheerfulness emerging as the most prominent theme. Several parents described high levels of enjoyment and enthusiasm associated with participation in gymnastics activities. For instance, one parent E-16 noted that the child enjoyed gymnastics to such an extent that it became a frequent topic of conversation across different settings. Similarly, another parent E-19 reported that the child typically appeared cheerful after gymnastics sessions due to enjoyment of the lessons. In addition, expressions of willingness and eagerness were highlighted, as one parent E-4 described the child becoming highly excited in anticipation of gymnastics classes and showing immediate readiness when reminded of the activity.

## Discussion

The absence of statistically significant differences in pretest scores between the experimental and control groups represents an expected and desirable outcome, indicating baseline comparability between the groups. This baseline equivalence may also be attributable to the children’s similar socio-cultural backgrounds. In contrast, the differences observed in posttest scores are more likely to reflect experiences gained during the implementation of the education program rather than pre-existing individual characteristics. From the perspective of Vygotsky’s sociocultural theory (1983; cited in [54]), child development cannot be considered independently of the social context in which learning occurs. Accordingly, this theoretical framework provides a basis for understanding how structured educational programs implemented during early childhood may influence social development processes through guided interaction and shared experiences.

Although both groups demonstrated improvements over time, the magnitude of change was more pronounced in the experimental group. While gains in the control group may be attributed to natural developmental processes and ongoing preschool education, the greater improvement observed in the experimental group suggests that the gymnastics intervention contributed additional developmental benefits.

The observed improvements in the control group, despite the absence of a specific intervention, can be explained by the continuous and dynamic nature of child development [55]. Accordingly, children’s social development in the control group likely progressed through ongoing participation in preschool education. Supporting this interpretation, previous research has shown that both the duration and quality of preschool education contribute positively to children’s social-emotional, cognitive, and social development [56, 57].

Posttest scores of children in the experimental group were significantly higher than those of children in the control group. This difference is likely attributable to the structured experiences provided within the scope of the basic gymnastics training program. The observed improvements in the experimental group following the intervention were anticipated; however, these changes should be interpreted not as the direct and isolated effects of gymnastics training, but rather as outcomes embedded within broader developmental processes. From a developmental perspective, development is holistic, with different domains mutually influencing one another [58, 59]. Accordingly, supporting motor development through gymnastics may be expected to exert concomitant effects on social and social-emotional development. This interaction among developmental domains aligns with the holistic perspective of child development, which posits that motor, language, cognitive, and social-emotional domains mutually influence one another during early childhood. A further explanation for the observed effects may lie in the enhanced sense of self-efficacy associated with participation in gymnastics training. Through repeated engagement in structured physical tasks, children experience mastery and success, which can strengthen their beliefs in their own abilities. Such experiences may foster a supportive context for psychological well-being by presenting challenges that are appropriately matched to children’s developmental levels. In turn, these experiences may contribute to the development of coping skills. Increased self-efficacy may then enable children to regulate their emotional responses more effectively in challenging situations and to cope more adaptively with experiences of disappointment. Evidence from prior research supports the association between physical activity and self-regulatory capacities in early childhood. For instance, a study conducted with children aged 5–6 years reported positive relationships between physical activity levels and multiple developmental outcomes, including self-regulation, adaptation, communication, adaptive behavior, autonomy, affect, and social interaction [60]. Consistent with these findings, Becker et al. [61] demonstrated that engagement in active play contributes positively to the development of children’s self-regulation skills. Furthermore, a meta-analysis indicated that approximately 67% of exercise-based intervention studies reported significant improvements in self-regulation, underscoring the potential efficacy of structured physical activity programs in supporting self-regulatory development [62]. Overall, these findings are broadly consistent with the results of the present study. One plausible explanation for the observed improvements is that gymnastics training provides a structured social interaction environment that may foster the development of self-regulation, emotion regulation, and social competence. Distinctive features of gymnastics, such as adherence to rules, sequential movement patterns, and structured group-based activities, differentiate it from many other forms of physical activity. Engaging in activities governed by explicit rules requires behaviors such as waiting in line, following instructions, and coordinating actions within a group, thereby promoting impulse control and socially appropriate behavior. This structured context may facilitate children’s ability to regulate their emotions within social settings.

Moreover, collaborative goal-oriented tasks inherent in gymnastics activities encourage children to work together with peers toward shared objectives. Such collaborative experiences are considered critical for the development of social adaptation and communication skills during early childhood. Through joint movement, peer observation, and cooperative efforts to achieve success, children are provided with opportunities to strengthen social bonds and regulate emotional responses within group contexts. Collectively, these processes may support the transfer of self-regulation, emotion regulation, and social competence skills from structured training environments to everyday life. Consistent with the present findings, Pushkina [12] reported that participation in rhythmic gymnastics training contributes to improvements in children’s discipline, time management, responsibility, cooperation, communication, patience, leadership, social confidence, and adaptability. These findings are consistent with the results of the present study and further suggest that the effects of gymnastics training should be evaluated in relation to the characteristics of the implementation process. Given that executive functions and self-regulation are closely interrelated constructs [63, 64], gymnastics training may have contributed to the observed improvements in self-regulation skills among children in the experimental group. During gymnastics activities, children are required to remember and follow instructions (working memory), inhibit or modify ongoing movements when necessary (inhibitory control), and flexibly switch between tasks (cognitive flexibility). Accordingly, the structured environment inherent in gymnastics training may indirectly enhance both emotion regulation and self-regulation skills by engaging and strengthening executive functions. Zhang et al. [65] reported that gymnastics training has a significant effect on preschool children’s executive functions, including response regulation and the management of cognitive conflicts. These findings are consistent with previous research demonstrating that participation in sports is associated with children’s mental and emotional development and contributes to improved emotional understanding [66]. Similarly, Vasilopoulos and Ellefson [67] highlighted a positive relationship between physical activity and emotion regulation skills during early childhood. In addition to supporting motor development, gymnastics training has been shown to promote cooperation, leadership, responsibility, self-confidence, and effective coping with stress in children [68].

In line with these findings, the combined quantitative and qualitative results of the present study indicate that children in the experimental group not only improved in structured physical tasks, such as progressing through activities and waiting in line, but also demonstrated notable gains in social-emotional skills, including patience, turn-taking, coping with disappointment, and the regulation of anger and anxiety. Qualitative findings complement the quantitative results by capturing observable changes in children’s behavioral patterns and emotional responses. Together, these findings support the notion that basic gymnastics training may exert differential effects on children’s emotion regulation skills. In addition, parental reports describing children’s ability to calm themselves, regulate the intensity of emotional responses, align emotions, thoughts, and behaviors with goal-directed actions, and influence others’ emotions and behaviors may be considered indicators of effective emotion regulation [69]. More specifically, these qualitative observations align particularly with the improvements observed in emotion regulation and social competence measures, reinforcing the interpretation that structured gymnastics activities facilitated both behavioural and emotional regulation processes.

Taken together, the qualitative and quantitative findings complement one another across the examined variables and underscore the multidimensional impact of the basic gymnastics education program.

## Conclusions

The findings of this study indicate that the Basic Gymnastics Training Program, implemented over an eight-week period, was associated with improvements in emotion regulation and social competence among children aged 48–69 months. Quantitative results demonstrated that children in the experimental group exhibited statistically significant increases in self-regulation, emotion regulation, and social competence compared to those in the control group. When interpreted in light of the post-intervention differences observed between groups, these findings suggest a potential relationship between participation in the program and positive changes in children’s self-regulatory and social-emotional skills. Given the relatively limited sample size, the results were evaluated not only in terms of statistical significance but also with respect to the magnitude of the observed effects. Between-group comparisons indicated that the magnitude of changes in the experimental group exceeded those observed in the control group, which may be considered indicative of the educational program’s contribution. These findings are further supported by the qualitative data, which indicate that gymnastics training exerts positive effects on a range of social-emotional outcomes, particularly making friends, expressing emotions, following rules, sharing, and displaying positive affect. The observation that some variables in the control group also demonstrated significant pretest–posttest changes is consistent with the developmental nature of early childhood and the influence of ongoing preschool education. However, the direction and magnitude of the changes observed in the experimental group were more pronounced, rendering the contribution of the educational program more evident. Collectively, these results suggest that gymnastics may serve as an effective approach for supporting skills central to social-emotional development in early childhood, including understanding and expressing emotions, managing emotional responses appropriately, engaging in role-play, adhering to rules, waiting for one’s turn, and coping with everyday challenges. These skills constitute core components of social-emotional development in early childhood and are closely associated with later academic adjustment and interpersonal relationships. Accordingly, the present findings indicate that gymnastics contributes not only to children’s physical development but also to their social-emotional development. This evidence further aligns with developmental approaches that emphasize the importance of adopting a holistic perspective on child development. The findings of the present study are consistent with existing literature, reinforcing the view that physical activities in early childhood support not only motor development but also social-emotional development. This consistency with previous research strengthens the credibility of the findings and suggests that comparable outcomes may be observed across different early childhood populations and contexts. Given its structured nature—characterized by explicit rules, turn-taking, instruction-following, and coordinated group movement—gymnastics training may be effectively integrated into early childhood education programs as a pedagogical tool. When incorporated into educational programs, gymnastics training can provide a structured environment in which children are regularly exposed to situations that require self-regulation and social competence, thereby supporting their developmental growth. Programs that include gymnastics activities may be implemented within play-based learning frameworks in weekly instructional schedules and explicitly linked to social-emotional development outcomes. The effectiveness of such programs is likely influenced by several implementation-related factors, including the trainer’s professional competence, the duration and intensity of the intervention, the characteristics and developmental profile of the participant group, and the contextual features of the environment in which the program is delivered. Specifying educational objectives that target skills such as emotion regulation, cooperation, turn-taking, and rule-following through gymnastics-based activities may enhance the effectiveness of early childhood education programs. In this context, it is recommended that gymnastics-based practices be systematically aligned with social-emotional learning objectives through structured in-service training programs for preschool teachers.

### Limitations and suggestions

Several limitations should be considered when interpreting the findings of this study. First, the relatively small sample size, comprising 20 children in the experimental group and 20 in the control group, may limit the statistical power and generalizability of the results. Second, all participating children were drawn from a middle socioeconomic background; children from lower and higher socioeconomic strata were not represented. This sample characteristic restricts the extent to which the findings can be generalized to broader and more socioeconomically diverse populations. Accordingly, caution is warranted when interpreting the findings across different socioeconomic and demographic contexts. One limitation of the study is that the outcome measures were based on teacher reports. Although these scales rely on natural classroom observations, teachers were aware of the group allocation, which may have introduced potential evaluation bias. Future studies may benefit from including blinded assessors or multiple data sources.

Such subjectivity may have influenced the interpretation of the findings. In addition, the instructor responsible for implementing the gymnastics program may have been aware of the content of the assessment scales, which could have influenced the implementation process and potentially affected the outcomes. These considerations suggest that the implementation process of the education program itself may have influenced the observed outcomes. An additional limitation is that the intervention was limited to an eight-week basic gymnastics program, and no follow-up or retention assessment was conducted to examine the persistence of the observed effects. Consequently, conclusions regarding the long-term sustainability of the changes observed in children should be interpreted with caution. Finally, the study was conducted within a single sociocultural context, which limits the generalizability of the findings to other cultural settings. Accordingly, the potential influence of cultural factors on children’s social-emotional development could not be examined within the scope of the present research.

Future research may examine the effects of basic gymnastics education programs among children who have experienced migration, natural disasters, or social disadvantage. Moreover, studies employing larger and more socioeconomically and culturally diverse samples could further explore the broader impact and generalizability of such programs. In this regard, longitudinal research designs would be particularly valuable for providing stronger evidence regarding the developmental trajectories and persistence of observed effects over time.

Longitudinal approaches may also allow for the examination of the effects of basic gymnastics education across different developmental domains and facilitate an evaluation of the long-term outcomes of these programs into later developmental periods. Tracking the sustainability of training-related gains would offer important insights into the enduring influence of gymnastics-based interventions.

From an applied perspective, integrating basic gymnastics programs into preschool education curricula under the supervision of qualified physical education teachers appointed by the Ministry of National Education may represent a critical step toward systematic and sustainable implementation. However, to rigorously evaluate the effectiveness of such practices, future studies should account for key implementation-related variables, including program fidelity, instructional quality, and contextual factors. Collectively, longitudinal and implementation-focused research will provide essential evidence regarding the long-term contributions of gymnastics-based interventions to child development.

## Supplementary Information

Supplementary Material 1.

## Data Availability

No datasets were generated or analysed during the current study.

## Appendix

**Table 5:** 8-week basic gymnastics training program

Week	Target Behavior	Event Content	Material / Note	Duration
1. Week	Group harmony, listening, taking turns	Warm-up games, getting to know each other in a circle, simple station games	Cushions, traffic cones	40 min (Warm-up: 5 min, Main activity: 25 min, Cool-down: 5 min, Emotional sharing: 5 min)
2. Week	Balance skill	Walking on a balance beam, overcoming obstacles	Balance board, cones	40 min (Warm-up: 5 min, Main activity: 25 min, Cool-down: 5 min, Emotional sharing: 5 min)
3. Week	Field awareness and orientation	Right-left direction exercises, foot coordination	Ring, colored string	40 min (Warm-up: 5 min, Main activity: 25 min, Cool-down: 5 min, Emotional sharing: 5 min)
4. Week	Waiting in line, being patient	Collect station change, skip in order	Small balls	40 min (Warm-up: 5 min, Main activity: 25 min, Cool-down: 5 min, Emotional sharing: 5 min)
5. Week	Confidence and self-confidence	Jumping on a mini trampoline, high jump movement	Trampoline, soft landing mat	40 min (Warm-up: 5 min, Main activity: 25 min, Cool-down: 5 min, Emotional sharing: 5 min)
6. Week	Collaborative work, cooperation	Pairwise ball carrying, transferring objects in groups	Plastic ball, basket	40 min (Warm-up: 5 min, Main activity: 25 min, Cool-down: 5 min, Emotional sharing: 5 min)
7. Week	Motor planning	Mini obstacle course: roll, jump, balance	Obstacle course equipment	40 min (Warm-up: 5 min, Main activity: 25 min, Cool-down: 5 min, Emotional sharing: 5 min)
8. Week	Autonomy and the emergence of your emotional expression	Free movement – “design your own movement”, closing ceremony	Music	40 min (Warm-up: 5 min, Main activity: 25 min, Cool-down: 5 min, Emotional sharing: 5 min)

**Table 6:** 8-week basic gymnastics training program scientific basis

Scale	Event Supported by the Program	Explanation
Self-Regulation Skills Scale	Waiting in line, following instructions	Sequential movement, attention control
Emotion Regulation Scale	Free movement, coping with failure	Expressing emotions, managing disappointment
Social Competence and Behavior Assessment Scale	Collaborative work, sharing	Collaboration, leadership tracking, compliance with rules
