# Supplementary material for: Supporting social-emotional and behavioral skills in preschool children through gymnastics
Source: BMC Pediatr. 2026 Jun 25;26:679. doi: 10.1186/s12887-026-07204-8 (PMC13393845; doi:10.1186/s12887-026-07204-8)
Supplement: Supplementary file 1 — Supplementary Material 1. [file 12887_2026_7204_MOESM1_ESM.pdf]

STROBE Statement—checklist of items that should be included in reports of observational studies

	Item No	Recommendation
**Title and abstract**	1	(*a*) Page 1 The mixed-method quasi-experimental design is clearly indicated in the Title and Abstract.
		(*b*) Page 1 Abstract section: Background, Methods, Results, and Conclusion are structured
**Introduction**		
Background/rationale	2	Pages 1-3 Introduction section: Theoretical framework and rationale are detailed
Objectives	3	Page 3 Research Questions and Hypotheses subsection
**Methods**		
Study design	4	Pages 3-4 Method section, Research Design subsection
Setting	5	Page 4 Method section, Research Design and Procedure subsections
Participants	6	(*a*) *Page 4 Method section, Study Group subsection: Inclusion criteria and group allocations*
		(*b*) *Page 4 Method section, Study Group subsection*
Variables	7	Pages 4-5 Method section, Data Collection Tools and Procedure subsections
Data sources/ measurement	8*	*Pages 4-5 Method section, Data Collection Tools subsection*
Bias	9	Page 4 Addressed through the use of intact classroom groups, temporal separation of intervention sessions, standardized data collection procedures, and independent administration of assessment tools.
Study size	10	Page 4 Method section, Study Group subsection: Sample size justified by G*Power 3.1 a priori power analysis
Quantitative variables	11	Page 5 (Method section, Data Analysis subsection)
Statistical methods	12	(*a*) Page 5 Method section, Data Analysis subsection
		(*b*) Page 5 Method section, Data Analysis subsection
		(*c*) N/A (No subgroup analyses were performed)
		(*d*) *N/A No missing data occurred; all 40 participants completed the study*
		(*e*) N/A

Continued on next page

**Results**		
Participants	13*	(a) Pages 5-6 Findings section, Quantitative Findings: Complete retention with zero attrition
		(b) Pages 5-6 Findings section, Quantitative Findings: All 40 participants completed the study
		(c) N/A
Descriptive data	14*	(a) N/A
		(b) Indicate number of participants with missing data for each variable of interest
Outcome data	15*	*Pages 5-6 (Findings section, presented thoroughly in Table 1 and Table 2)*
Main results	16	(*a*) Pages 5-6 Findings section, statistical significance and effect sizes reported in Table 1 and Table 2
		(*b*) N/A
		(*c*) N/A
Other analyses	17	Pages 6-7 Findings section, Qualitative Findings subsection: Triangulated with parent interview themes in Table 3 and Table 4
**Discussion**		
Key results	18	Pages 7-8 Discussion section, first paragraph summarizing the main intervention effects
Limitations	19	Page 9 Discussion section, Limitation's paragraph
Interpretation	20	Pages 8-9 Discussion section, integrated with Vygotsky's sociocultural theory and current literature
Generalisability	21	Page 9 Discussion section, Conclusion and Implications paragraphs
**Other information**		
Funding	22	Page 10 Declarations section, Funding statement

*Give information separately for cases and controls in case-control studies and, if applicable, for exposed and unexposed groups in cohort and cross-sectional studies.

Note: An Explanation and Elaboration article discusses each checklist item and gives methodological background and published examples of transparent reporting. The STROBE checklist is best used in conjunction with this article (freely available on the Web sites of PLoS Medicine at http://www.plosmedicine.org/, Annals of Internal Medicine at http://www.annals.org/, and Epidemiology at http://www.epidem.com/). Information on the STROBE Initiative is available at www.strobe-statement.org.
